# The Relationship of Retinal Vessel Diameters and Fractal Dimensions with Blood Pressure and Cardiovascular Risk Factors

**DOI:** 10.1371/journal.pone.0106551

**Published:** 2014-09-04

**Authors:** Pengli Zhu, Feng Huang, Fan Lin, Qiaowei Li, Yin Yuan, Zhonghai Gao, Falin Chen

**Affiliations:** 1 Department of Geriatric Medicine, Fujian Provincial Hospital, Fujian Provincial Institute of Clinical Geriatrics, Fujian Medical University, Fuzhou, China; 2 Department of Ophthalmology, Fujian Provincial Hospital, Fuzhou, China; 3 Clinical Laboratory Center, Fujian Provincial Hospital, Fuzhou, China; University of Campinas, Brazil

## Abstract

**Background:**

This study aimed to investigate the correlation between quantitative retinal vascular parameters such as central retinal arteriolar equivalent (CRAE) and retinal vascular fractal dimension (D(f)), and cardiovascular risk factors in the Chinese Han population residing in the in islands of southeast China.

**Methodology/Principle Findings:**

In this cross-sectional study, fundus photographs were collected and semi-automated analysis software was used to analyze retinal vessel diameters and fractal dimensions. Cardiovascular risk factors such as relevant medical history, blood pressure (BP), lipids, and blood glucose data were collected. Subjects had a mean age of 51.9±12.0 years and included 812 (37.4%) males and 1,357 (62.6%) females. Of the subjects, 726 (33.5%) were overweight, 226 (10.4%) were obese, 272 (12.5%) had diabetes, 738 (34.0%) had hypertension, and 1,156 (53.3%) had metabolic syndrome. After controlling for the effects of potential confounders, multivariate analyses found that age (*β* = 0.06, *P* = 0.008), sex (*β* = 1.33, *P* = 0.015), mean arterial blood pressure (*β* = −0.12, *P*<0.001), high-sensitivity C-reactive protein (*β* = −0.22, *P* = 0.008), and CRVE (*β* = 0.23, *P*<0.001) were significantly associated with CRAE. Age (*β* = −0.0012, *P*<0.001), BP classification (prehypertension: *β* = −0.0075, *P* = 0.014; hypertension: *β* = −0.0131, *P* = 0.002), and hypertension history (*β* = −0.0007, *P* = 0.009) were significantly associated with D(f).

**Conclusions/Significance:**

D(f) exhibits a stronger association with BP than CRAE. Thus, D(f) may become a useful indicator of cardiovascular risk.

## Introduction

Retinal vessels are the only segment of the human microcirculation that can be observed directly. Recently, the associations between central retinal arteriolar equivalent (CRAE) and many cardiovascular risk factors have been demonstrated [1]–[5]. The American Heart Association estimates that more than 1 in 3 adults Americans have one or more types of cardiovascular disease [6]. Because of this high prevalence, diagnostic screening tools are important in order to optimally manage the disease. Retinal vasculature changes associated with hypertension can generally be divided into three groups: 1) classic retinal vascular changes in response to blood pressure (hypertensive retinopathy), 2) changes in retinal vascular caliber, and 3) changes in global geometric patterns of the retina [7].

Quantitative assessment of retinal vascular parameters is a precise and reliable method of investigating the relationship between microcirculation and cardiovascular disease [8], and most studies have focused on retinal vascular caliber, which is correlated with blood pressure (BP) [9]. Pooled data from the Beaver Dam Eye Study and the Blue Mountains Eye Study demonstrated that smaller arterioles and larger venules predicted an increased risk of mortality from coronary heart disease [10]. A larger central retinal vein is associated with smoking, low high-density lipoprotein cholesterol (HDL-C), elevated glycated hemoglobin (HbA_1c_), and high body mass index (BMI), and is able to independently predict the 6-year incidence of hypertension [5], [11]. A recent study conducted over an 18-year period found that every 10 mm Hg increase in mean systolic blood pressure (SBP) was associated with a 1.9 reduction in CRAE [12].

The human retinal vascular network, including its branching pattern, has been demonstrated to have a fractal structure [13], [14]. Retinal vascular fractal dimension, D(f), has been shown to be related to hypertension [8], [9], [15], diabetic retinopathy [16], [17], chronic kidney disease (CKD) [18], stroke [19], [20], and mortality from coronary heart disease [21]. Retinal vessel diameters have been shown to vary in different populations [5], [22], and there is a paucity of data on the correlation between changes in the retinal vasculature and risk factors for cardiovascular disease in the native Chinese population [22], [23]. In addition, D(f) has not been described in the Chinese population. The coastal regions of China have a high prevalence of fishery workers, and studies of fishery workers in other countries suggest a higher prevalence of hypertension in these individuals [24], [25].

The current cross-sectional study was designed to investigate the relationship of quantitative retinal vascular parameters such as CRAE, central retinal venular equivalent (CRVE), and D(f) with cardiovascular risk factors in a Chinese Han population residing in the islands off southeast China.

## Methods

### Subjects

The villages of Tailu, Beijiao, and Xiubang in the Tailu township in Lienchiang county and Kungtung, Kunghsi, Yantai, and Wenwo in Haidao township in Xiapu county in Fujian Province were randomly selected for this cross-sectional investigation, which took place from July, 2011 to November, 2011. These participants were selected because it has been shown that coaster villagers are at higher risk for hypertension [26].

Inclusion criteria were voluntary participants aged 30 years or more living in Tailu and Haidao townships for more than 5 years. Subjects were excluded if they were diagnosed with or were suspected to have secondary hypertension, acute myocardial infarction, acute stroke, infection, or an inflammatory disease. Individuals who had persistent arrhythmias (e.g., atrial fibrillation), chronic heart dysfunction, pulmonary hypertension, acute and chronic infectious diseases, malignancy, hematuria or urinary tract infection, peripheral vascular disease, ankle-brachial index (ABI) <0.6, or paralysis were also excluded. In addition, individual with a history of serious eye diseases such as corneal diseases, glaucoma, macular degeneration, or eye injuries, pregnant women, and anyone who could not cooperate with study requirements were excluded.

All participants provided signed informed consent form. This study was conducted in accordance with the Declaration of Helsinki, and was approved by the Institutional Review Board of the Fujian Provincial Hospital.

### Data collection

Age, educational background, smoking history, alcohol history, and relevant medical history (e.g. hypertension, chronic heart failure and arrhythmia) were obtained using questionnaires. Physical examinations included height (cm), weight (kg), heart rate (HR, beats/minute) and BP. Examinees could not smoke or drink coffee within 30 minutes before measuring blood pressure. After emptying his/her bladder and resting in a quiet environment for 5 to 10 minutes, the examinee’s BP was measured by using a standard vertical mercury sphygmomanometer on the right upper arm. The average of three BP measurements obtained 1–2 minutes apart was calculated and recorded.

### Classification definitions

BMI was calculated as weight in kilograms divided by height in meters squared (kg/m^2^). According to the BMI, subjects were divided in three categories: normal or underweight (BMI<24 kg/m^2^), overweight (24–28 kg/m^2^) and obese (>28 kg/m^2^) [27]. Hypertension was defined as SBP≥140 mm Hg and diastolic BP (DBP) ≥90 mm Hg in the presence or absence of anti-hypertension medications [28]. In the absence of anti-hypertensive medications, a systolic BP of 120–139 mm Hg and diastolic BP of 80–89 mm Hg was defined as “prehypertension” and a systolic BP of <120 mm Hg and diastolic BP of <80 mm Hg was defined as normal [29].

### Laboratory testing

After an 8-hour overnight fast, blood samples were obtained measurement of triglyceride (TG), total cholesterol (TC), HDL-C, low-density lipoprotein cholesterol (LDL-C), fasting plasma glucose (FPG), HbA_1c_, high-sensitivity C-reactive protein (hs-CRP), and serum uric acid. Participants diagnosed with diabetes, those currently taking anti-diabetic medications, or with an HbA_1c_≥6.5% were defined as diabetic [29].

### Data collection and quantitative analysis of retinal vessels

Digital fundus photographs were obtained using non-mydriatic fundus photography. Binocular digital photographs were taken using a 45° high-resolution fundus camera (Topcon NW-8, Topcon Corp, Tokyo, Japan; Nikon D90, Nikon Corp, Tokyo, Japan). Retinal fundus images were centered on the optic disc. The side of fundus photograph with a better quality image was analyzed, and double-blinded analysis was performed by two professionally trained ophthalmologists.

Semi-automated software (Singapore I Vessel Assessment, Version 3.0 [SIVA]; Exploit Technologies Private Limited, Singapore) was used for the quantitative analysis. SIVA software was been developed in collaboration with the Singapore Eye Research Institute, and has the capability of measuring a number of retinal vessel parameters including arteriolar/venular caliber, tortuosity, branching angles, and fractals.

The SIVA software was per the developer’s protocol. The software employs automatic optic disc detection and measures caliber and geometry of peripheral vessels up to 2 DD from the optic disc margin. Measurements were taken within a concentric grid (0.5–2.0 DD) centered on the optic disc [30]. The program automatically traced and identified all vessels (artery or vein) within the concentric circular grid, thus generating a skeleton image of the retinal microvasculature. The spatial resolution of each image was 3216×2136 pixels, and the images were stored without compression before analysis. The size of the scaling window was 1366×731 pixels. With the box counting method, the digitized retinal image was divided into a large number of equally sized square boxes, and the number of boxes containing a section of the refined skeletonized line tracing is counted; the process is then repeated with a different sized box. The fractal dimension of the skeletonized line tracing is the slope of the line obtained via plotting the logarithm of the number of boxes through which the tracing passes against the logarithm of the size of the boxes. Trained graders then correctly identified all vessels traced and removed all artifacts from the fundus photographs that may have been incorrectly drawn as vessels from the tracing. The reproducibility of this method has been shown in several studies [9], [15], [30].

**Figure 1:**
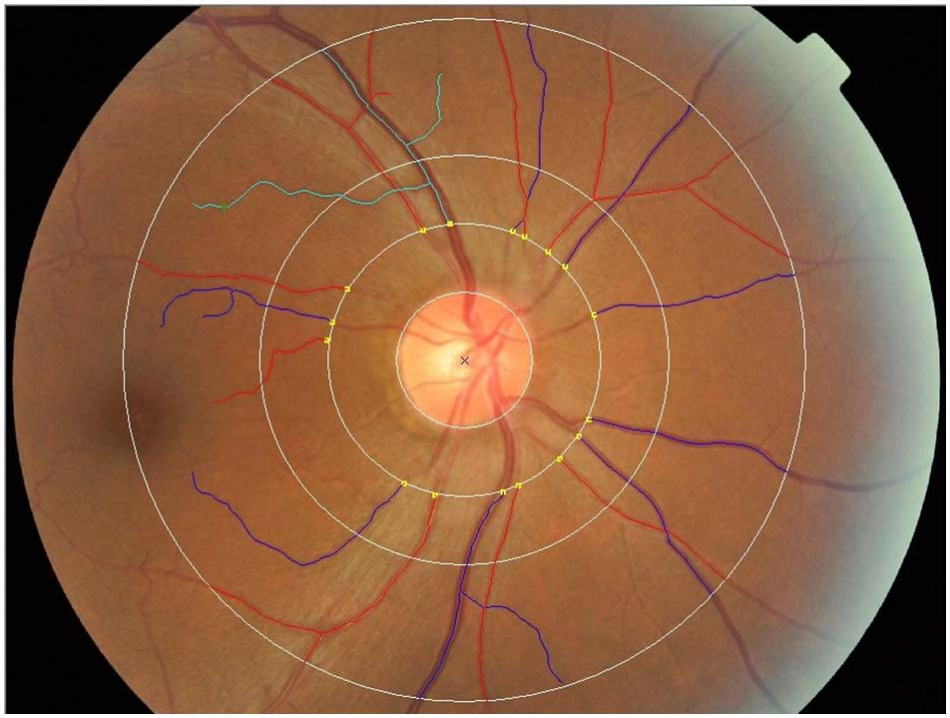
Fundus photographs processed by SIVA version 3.0 software. Test zone was 0.5- to 2.0-disc diameters away from the optic disc margin. A red line represents an artery and a blue line represents a vein. Fractal dimension, D(f), is a complexity indicator determined by reactive vessels obtained from vascular tracking through the box counting dimension method.

CRAE and CRVE measurements, performed 0.5- to 1.0-disc diameter away from the optic disc margin, were calculated by the revised Knudtson-Parr-Hubbard formula (Fig. 1) [31]. Fractal geometry can be used to quantify a branching pattern that exhibits self-similarity, and the retinal vasculature has a fractal-like architecture. D(f), which is a measure of a fractal structure characterizing the distribution of the branching vascular system in two-dimensional space, was used to quantify the branching pattern of the retinal blood vessels. Retinal vascular D(f) was calculated from a skeletonized line tracing using the box-counting method, which divides each digital photograph into a series of squares of various side lengths, and the number of boxes is counted [8], [15]. D(f) was defined as the gradient of logarithms of the number of boxes and the size of the boxes [9]. Larger values indicate a more complex branching pattern, and two-dimensional fractal dimensions are between 1 and 2. D(f) measurements were performed 0.5- to 2.0-disc diameters away from the optic disc margin, as described by Cheung et al. [8].

### Statistical analysis

Continuous variables were presented as mean ± standard deviation, and categorical variables by count and percentage. To investigate the potential confounders associated with the retinal vascular parameters, simple and multiple linear regressions were performed. In univariate analyses, all significant factors were selected for entry into the multivariate models, except for those that had collinearity with other variables. Statistical analyses were performed with SAS software version 9.2 (SAS Institute Inc., Cary, NC). A two-sided P value<0.05 was considered to be statistically significant.

## Results

Based on the household registration in the surveyed area, there were 8,947 who were more than 30 years of age. Of these, 4,616 were selected according to the age stratification theory and were issued study invitations. Of these, 3,614 received the study invitations because some people had relocated. A total of 3,343 people participated in the survey; therefore the response rate was 92.5%. A total of 1,174 subjects were excluded (421 with incomplete data, 49 with infectious diseases, 14 with atrial fibrillation, 15 with an ABI<0.6, 675 whose fundus photographs were inadequate); thus, 2,169 subjects were included in the analysis.

Baseline characteristics of the included and excluded subjects are shown in Table 1, and demographic and clinical data of the 2,169 participants are shown in Table 2. Study participants had a mean age of 51.9±12.0 years, and included 812 (37.4%) males and 1357 (62.6%) females. Among these subjects, 1,217 (56.1%) had a normal BMI, 726 (33.5%) were overweight, and 226 (10.4%) were obese. According to the JNC 7 criteria, 754 (34.8%) subjects had normal BP, 596 (27.5%) had prehypertension, and 819 (37.8%) had stage 1 or stage 2 hypertension.

**Table 1:** Characteristics of the included and excluded subjects (N = 3,343).

	Excluded	Included	
	(*n* = 1,174)	(*n* = 2,169)	*P*
Age, y	57.3±14.5	51.9±11.9	**<0.001** *
Males, %	524 (44.6)	812 (37.4)	**0.001** *
BMI, kg/m^2^	23.9±2.9	23.8±3.5	0.287
SBP, mmHg	134.4±25.9	127.0±21.9	**0.001** *
DBP, mmHg	80.2±12.8	78.6±11.9	0.322
MAP, mmHg	98.2±15.6	94.7±14.1	**0.021** *
PP, mm Hg	54.2±19.9	48.4±15.9	**<0.001** *
HR, beats per minute	71.3±9.7	71.2±9.3	0.925
Smoking, %	306 (26.1)	1828 (84.3)	**<0.001** *
Alcohol consumption, %	246 (21.0)	341 (15.7)	**<0.001** *
Diabetes, %	183 (15.6)	272 (12.5)	**0.016** *
Hypertension, %	568 (48.4)	819 (37.8)	**<0.001** *
TG, mmol/L	1.2±0.9	0.9±0.5	**<0.001** *
TC, mmol/L	5.1±1.1	5.0±1.1	0.408
HDL-C, mmol/L	1.2±0.4	1.2±0.3	0.300
LDL-C, mmol/L	2.8±0.9	2.8±0.9	0.792
FPG, mmol/L	5.5±2.2	5.3±1.5	**0.026** *
HbA_1c_, %	5.8±0.9	5.7±0.7	**0.001** *
Uric acid, µmol/L	324.3±92.1	315.1±84.8	**0.004** *
hs-CRP, mg/L	1.9±4.7	1.4±3.4	**0.001** *

Continuous variables were reported as mean ± deviation (SD) and categorical variables as percentages (%).

SBP, systolic blood pressure; DBP, diastolic blood pressure; MAP, mean arterial pressure; HR, heart rate; BMI, body mass index; TG, triglyceride; TC, total cholesterol; HDL-C, high density lipoprotein cholesterol; LDL-C, low density lipoprotein cholesterol; FPG, fasting plasma glucose; HbA_1c_, glycated hemoglobin; hs-CRP, high-sensitivity C-reactive protein.

*P<0.05 indicates a significant difference between groups.

**Table 2:** Characteristics of study subjects (n = 2,169).

Age, y	51.9±12.0
Gender	
Males	812 (37.4)
Females	1357 (62.6)
BMI, kg/m^2^	
<24	1217 (56.1)
24–28	726 (33.5)
>28	226 (10.4)
SBP, mm Hg	126.9±21.9
DBP, mm Hg	78.6±11.9
MAP, mm Hg	94.7±14.1
PP, mm Hg	48.4±15.9
Blood pressure classification	
Normal	754 (34.8)
Prehypertension	596 (27.5)
Hypertension	819 (37.8)
HR, beats/minute	71.2±9.3
Smoking	
Never	1754 (80.9)
Current or former	415 (19.1)
Alcohol consumption	
Never	1828 (84.3)
Current or former	341 (15.7)
Diabetes	
Absent	1897 (87.5)
Present	272 (12.5)
Hypertension	
Absent	1431 (66.0)
Present	738 (34.0)
Metabolic syndrome	
Absent	1013 (46.7)
Present	1156 (53.3)
TG, mmol/L	0.9±0.5
TC, mmol/L	5.0±1.0
HDL-C, mmol/L	1.2±0.3
LDL-C, mmol/L	2.8±0.9
FPG, mmol/L	5.3±1.5
Uric acid, µmol/L	315.0±84.8
HbA_1c_, %	5.7±0.7
hs-CRP, mg/L	1.4±3.4
CRAE, µm	134.1±11.1
CRVE, µm	184.0±16.6
D(f)	1.4±0.1

Continuous variables were reported as mean ± deviation (SD) and categorical variables as count (%).

BMI, body mass index; SBP, systolic blood pressure; DBP, diastolic blood pressure; MAP, mean arterial pressure; PP, pulse pressure; HR, heart rate; TG, triglyceride; TC, total cholesterol; HDL-C, high-density lipoprotein cholesterol; LDL-C, low-density lipoprotein cholesterol; FPG, fasting plasma glucose; HbA_1c_, glycated hemoglobin; hs-CRP, high-sensitivity C-reactive protein; CRAE, central retinal arteriolar equivalent; CRVE, central retinal venular equivalent; D(f), retinal vascular fractal dimension.

Hypertension: SBP≥140 mm Hg and DBP≥90 mm Hg, with or without medication. This group (*n* = 819) included subjects who were previously diagnosed (*n* = 738) and those diagnosed in this study based on the criteria above (*n* = 81).

Pre-hypertension: SBP 120–139 mm Hg and DBP 80–89 mm Hg without medication. Normal: SBP<120 mm Hg and DBP<80 mm Hg. Hypertension history was self-reported based on the questionnaire.

The mean CRAE, CRVE, and D(f) were 134.1±11.1 µm, 184.0±16.6 µm, and 1.4±0.1 µm, respectively. Simple linear regression analysis showed that age, gender, BMI, SBP, DBP, mean arterial pressure (MAP), pulse pressure (PP), BP classification, alcohol consumption, hypertension, TG, TC, LDL-C, hs-CRP, and CRVE were significantly associated with CRAE (all *P*≤0.041) (Table 3). After controlling for effects of potential confounders, multivariate analysis showed that age (*β* = 0.06, 95% confidence interval [CI] = 0.02 to 0.10, *P* = 0.008), sex (*ß* = 1.33, 95% CI = 0.26 to 2.40, *P* = 0.015), MAP (*ß* = −0.12, 95% CI = −0.17 to −0.07, *P*<0.001), hs-CRP (*ß* = −0.55, 95% CI = −0.96 to −0.15, *P* = 0.008) and CRVE (*ß* = 0.23, 95% CI = 0.20 to 0.25, *P*<0.001) were significantly associated with CRAE. Simple linear regression analysis also showed that age, SBP, DBP, MAP, PP, BP classification, smoking, alcohol consumption, hypertension, TG, uric acid, hs-CRP, and CRAE were significantly associated with CRVE (all, *P*≤0.021) (Table 4). After controlling for the effects of potential confounders, multivariate analysis showed that age (*ß* = −0.12, 95% CI = −0.19 to −0.05, *P*<0.001), smoking (*ß* = 2.87, 95% CI = 1.00 to 4.74, *P* = 0.003), and CRAE (*ß* = 0.51, 95% CI = 0.45 to 0.57, *P*<0.001) were significantly associated with CRVE.

**Table 3:** Simple and multiple linear regression analyses for CRAE.

	Univariate		Multivariate	
	*β* (95% CI)	*P* value	*β* (95% CI)	*P* value
Age, y	−0.05 (−0.09, −0.01)	**0.009** *	0.06 (0.02, 0.10)	**0.008** *
Gender				
Females (ref: males)	2.12 (1.16, 3.09)	**<0.001** *	1.33 (0.26, 2.40)	**0.015** *
BMI, kg/m^2^				
24–28 (ref: <24)	−1.83 (−2.85, −0.81)	**<0.001** *	−0.63 (−1.61, 0.35)	0.205
>28 (ref: <24)	−2.16 (−3.73, −0.58)	**0.007** *	−0.22 (−1.75, 1.32)	0.782
SBP, mm Hg	−0.09 (−0.11, −0.07)	**<0.001** *		
DBP, mm Hg	−0.17 (−0.21, −0.13)	**<0.001** *		
MAP, mm Hg	−0.15 (−0.18, −0.12)	**<0.001** *	−0.12 (−0.17, −0.07)	**<0.001** *
PP, mm Hg	−0.07 (−0.10, −0.04)	**<0.001** *	0.02 (−0.02, 0.05)	0.345
Blood pressure classification				
Prehypertension (ref: normal)	−2.28 (−3.46, −1.09)	**<0.001** *	−0.42 (−1.72, 0.88)	0.53
Hypertension (ref: normal)	−3.98 (−5.07, −2.89)	**<0.001** *	−0.43 (−2.28, 1.41)	0.645
HR, beats per minute	0.03 (−0.02, 0.08)	0.271		
Smoking				
Current or former (ref: never)	−0.35 (−1.54, 0.84)	0.564		
Alcohol consumption				
Current or former (ref: never)	−2.12 (−3.41, −0.84)	**0.001** *	−1.01 (−2.41, 0.39)	0.157
Diabetes				
Present (ref: absent)	−0.03 (−1.45, 1.38)	0.962		
Hypertension				
Present (ref: absent)	−1.77 (−2.76, −0.79)	**<0.001** *	0.14 (−0.99, 1.27)	0.807
Metabolic syndrome				
Present (ref: absent)	−0.72 (−1.66, 0.21)	0.131		
TG, mmol/L	1.29 (0.36, 2.22)	**0.007** *	0.60 (−0.30, 1.50)	0.195
TC, mmol/L	−0.47 (−0.91, −0.02)	**0.041** *	−0.31 (−1.02, 0.39)	0.384
HDL-C, mmol/L	−0.26 (−1.68, 1.16)	0.719		
LDL-C, mmol/L	−0.67 (−1.2, −0.14)	**0.013** *	0.15 (−0.69, 0.98)	0.731
FPG, mmol/L	−0.17 (−0.48, 0.14)	0.281		
Uric acid, µmol/L	0.004 (−0.002, 0.009)	0.218		
HbA_1c_, %	−0.13 (−0.82, 0.56)	0.707		
hs-CRP, mg/L†	−1.21 (−1.60, −0.82)	**<0.001** *	−0.55 (−0.96, −0.15)	**0.008** *
CRVE, µm	0.52 (0.46, 0.58)	**<0.001** *	0.23 (0.2, 0.25)	**<0.001** *

BMI, body mass index; SBP, systolic blood pressure; DBP, diastolic blood pressure; MAP, mean arterial pressure; PP, pulse pressure; HR, heart rate; TG, triglyceride; TC, total cholesterol; HDL-C, high-density lipoprotein cholesterol; LDL-C, low-density lipoprotein cholesterol; FPG, fasting plasma glucose; HbA_1c_, glycated hemoglobin; hs-CRP, high-sensitivity C-reactive protein; CRAE, central retinal arteriolar equivalent; CRVE, central retinal venular equivalent; D(f), retinal vascular fractal dimension.

*β* (95% CI): regression coefficient and 95% confidence interval.

*P<0.05 indicates a significant association with CRAE.

† Log transformation was applied.

The R^2^ for the multivariable regression model was 0.1621.

**Table 4:** Simple and multiple linear regression analyses for CRVE.

	Univariate		Multivariate	
	*β* (95% CI)	*P* value	*β* (95% CI)	*P* value
Age, y	−0.14 (−0.2, −0.08)	**<0.001** *	−0.12 (−0.19, −0.05)	**<0.001** *
Gender				
Females (ref: Males)	−0.84 (−2.29, 0.60)	0.253		
BMI, kg/m^2^				
24–28 (ref: <24)	−0.76 (−2.29, 0.77)	0.330		
>28 (ref: <24)	−1.91 (−4.27, 0.45)	0.112		
SBP, mm Hg	−0.07 (−0.10, −0.04)	**<0.001** *		
DBP, mm Hg	−0.08 (−0.14, −0.02)	**0.009** *		
MAP, mm Hg	−0.09 (−0.14, −0.04)	**<0.001** *	0.002 (−0.08, 0.08)	0.969
PP, mm Hg	−0.09 (−0.14, −0.05)	**<0.001** *	−0.04 (−0.09, 0.02)	0.18
Blood pressure classification				
Prehypertension (ref: normal)	−0.44 (−2.22, 1.34)	0.627	1.34 (−0.61, 3.30)	0.179
Hypertension (ref: normal)	−2.62 (−4.26, −0.98)	**0.002** *	1.89 (−0.89, 4.67)	0.182
HR, beats per minute	0.02 (−0.06, 0.09)	0.683		
Smoking				
Current or former (ref: never)	3.60 (1.83, 5.37)	**<0.001** *	2.87 (1.00, 4.74)	**0.003** *
Alcohol consumption				
Current or former (ref: never)	2.26 (0.34, 4.18)	**0.021** *	1.96 (−0.06, 3.98)	0.301
Diabetes				
Present (ref: absent)	1.03 (−1.08, 3.15)	0.337		
Hypertension			−0.89 (−2.59, 0.80)	0.301
Present (ref: absent)	−2.59 (−4.06, −1.11)	**0.001** *		
Metabolic syndrome				
Present (ref: absent)	0.77 (−0.63, 2.17)	0.283		
TG, mmol/L	2.56 (1.17, 3.95)	**<0.001** *	−0.10 (−0.75, 0.54)	0.751
TC, mmol/L	−0.44 (−1.11, 0.23)	0.195		
HDL-C, mmol/L	−2.10 (−4.21, 0.02)	0.052		
LDL-C, mmol/L	−0.78 (−1.57, 0.01)	0.052		
FPG, mmol/L	0.21 (−0.25, 0.68)	0.372		
Uric acid, µmol/L	0.012 (0.004, 0.021)	**0.003** *	0.01 (0.0002, 0.02)	**0.044** *
HbA_1c_, %	0.64 (−0.39, 1.66)	0.225		
hs-CRP, mg/L†	−0.87 (−1.46, −0.28)	**0.004** *	−0.07 (−0.68, 0.54)	0.824
CRAE, µm	0.23 (0.21, 0.26)	**<0.001** *	0.51 (0.45, 0.57)	**<0.001** *

BMI, body mass index; SBP, systolic blood pressure; DBP, diastolic blood pressure; MAP, mean arterial pressure; PP, pulse pressure; HR, heart rate; TG, triglyceride; TC, total cholesterol; HDL-C, high-density lipoprotein cholesterol; LDL-C, low-density lipoprotein cholesterol; FPG, fasting plasma glucose; HbA_1c_, glycated hemoglobin; hs-CRP, high-sensitivity C-reactive protein; CRAE, central retinal arteriolar equivalent; CRVE, central retinal venular equivalent; D(f), retinal vascular fractal dimension.

*β* (95% CI): regression coefficient and 95% confidence interval.

*P<0.05 indicates a significant association with CRVE.

† Log transformation was applied.

The R^2^ for the multivariable regression model was 0.1412.

The average D(f) was 1.365 (range, 1.123 and 1.515). Simple linear regression analysis showed that age, BMI, SBP, DBP, MAP, PP, BP classification, diabetes, hypertension, metabolic syndrome, TG, TC, LDL-C, HbA_1c_, and hs-CRP were significantly associated with D(f) (all, *P*≤0.010) (Table 5). After controlling for potential confounders, multivariate analysis showed that age (*ß* = −0.0012, 95% CI = −0.0014 to −0.001, *P*<0.001), BP classification (prehypertension: *ß* = −0.0075, 95% CI = −0.0134 to −0.0015, *P* = 0.014; hypertension: *ß* = −0.0131, 95% CI = −0.0216 to −0.0047, *P* = 0.002), and history of hypertension (*ß* = −0.0007, 95% CI = −0.0121 to −0.0018, *p* = 0.009) were significantly associated with D(f).

**Table 5:** Simple and multiple linear regression analyses for D(f).

	Univariate		Multivariate	
	*β* (95% CI)	*P* value	*β* (95% CI)	*P* value
Age, y	−0.0018 (−0.0019, −0.0016)	**<0.001** *	−0.0012 (−0.0014, −0.001)	**<0.001** *
Gender				
Females (ref: males)	0.0033 (−0.0013, 0.0078)	0.157		
BMI, kg/m^2^				
24–28 (ref: <24)	−0.0081 (−0.0129, −0.0033)	**0.001** *	0.0024 (−0.0026, 0.0074)	0.349
>28 (ref: <24)	−0.0172 (−0.0246, −0.0098)	**<0.001** *	0.0001 (−0.0076, 0.0078)	0.976
SBP, mm Hg	−0.0008 (−0.0009, −0.0007)	**<0.001** *		
DBP, mm Hg	−0.0010 (−0.0012, −0.0008)	**<0.001** *		
MAP, mm Hg	−0.0011 (−0.0013, −0.0010)	**<0.001** *	−0.0002 (−0.0004, 0)	0.105
PP, mm Hg	−0.0010 (−0.0011, −0.0009)	**<0.001** *	−0.0001 (−0.0003, 0)	0.151
Blood pressure classification				
Prehypertension (ref: Normal)	−0.0188 (−0.0241, −0.0136)	**<0.001** *		
Hypertension (ref: Normal)	−0.0427 (−0.0476, −0.0378)	**<0.001** *	−0.0075 (−0.0134, −0.0015)	**0.014** *
HR, beats per minute	−0.0001 (−0.0003, 0.0001)	0.432	−0.0131 (−0.0216, −0.0047)	**0.002** *
Smoking				
Current or former (ref: Never)	−0.0009 (−0.0065, 0.0047)	0.746		
Alcohol consumption				
Current or former (ref: Never)	−0.0019 (−0.0079, 0.0042)	0.540		
Diabetes				
Present (ref: Absent)	−0.0186 (−0.0252, −0.0120)	**<0.001** *	−0.0029 (−0.0093, 0.0035)	0.376
Hypertension				
Present (ref: Absent)	−0.0291 (−0.0336, −0.0246)	**<0.001** *	−0.007 (−0.0121, −0.0018)	**0.009** *
Metabolic syndrome				
Present (ref: Absent)	−0.0108 (−0.0152, −0.0065)	**<0.001** *	−0.0024 (−0.0075, 0.0028)	0.363
TG, mmol/L	0.0084 (0.0040, 0.0127)	**<0.001** *	0.0026 (−0.0017, 0.0069)	0.243
TC, mmol/L	−0.0038 (−0.0059, −0.0017)	**<0.001** *	0.003 (−0.0002, 0.0062)	0.07
HDL-C, mmol/L	−0.0018 (−0.0085, 0.0049)	0.597		
LDL-C, mmol/L	−0.0071 (−0.0095, −0.0046)	**<0.001** *	−0.0037 (−0.0075, 0.0002)	0.062
FPG, mmol/L	−0.0014 (−0.0029, 0.0001)	0.061		
Uric Acid, µmol/L	−0.00002 (−0.0001, 0.000003)	0.083		
HbA_1c_, %	−0.0123 (−0.0155, −0.0091)	**<0.001** *		
hs-CRP, mg/L†	−0.0084 (−0.0102, −0.0066)	**<0.001** *	−0.0005 (−0.0024, 0.0013)	0.563

BMI, body mass index; SBP, systolic blood pressure; DBP, diastolic blood pressure; MAP, mean arterial pressure; PP, pulse pressure; HR, heart rate; TG, triglyceride; TC, total cholesterol; HDL-C, high-density lipoprotein cholesterol; LDL-C, low-density lipoprotein cholesterol; FPG, fasting plasma glucose; HbA_1c_, glycated hemoglobin; hs-CRP, high-sensitivity C-reactive protein; CRAE, central retinal arteriolar equivalent; CRVE, central retinal venular equivalent; D(f), retinal vascular fractal dimension.

*β* (95% CI): regression coefficient and 95% confidence interval.

*P<0.05 indicates a significant association with D(f).

† Log transformation was applied.

The R^2^ for the multivariable regression model was 0.2031.

The Pearson’s correlation coefficient between PP and MAP was 0.573 (*P*<0.001). The variance inflation factors (VIFs) of MAP and PP were 2.785 and 1.782 in the CRAE model; 2.790 and 1.772 in the CRVE model; 2.731 and 2.755 in the D(f) model. VIF indicates the presence of multicollinearity; a VIF≥10 is often regarded as a sign of serious multicollinearity correction.

We compared FPG and diabetes in the univariate model and found no significant effect. If a variable had shown significance in the univariate model, it would have been included in the multivariate model. As seen in Tables 3, 4, and 5 FPG was not a significant factor in the univariate model, suggesting that FPG levels did not correlate with outcomes; therefore, that term was not included in the multivariate model for further analysis.

## Discussion

### Relationship of retinal vessel parameters with cardiovascular risk factors

A previous study reported a correlation between CRAE and plasmas TG [32]. The current study found that a narrow CRAE was associated with obesity and gender, while a large CRAE was associated age, smoking, and elevated HbA_1c_, consistent with previous reports [2]–[5], [33], [34]. The current study also showed that a decreased CRVE was associated with an increase of LDL-C; however, this correlation was not significant after adjusting for other cardiovascular risk factors. Therefore, we could not conclude that CRVE was associated with lipid levels. Our study did not find an association between CRVE and hs-CRP, consistent with the Cardiovascular Health Study [35], but inconsistent with the Beaver Dam [31] and multi-ethnic study of atherosclerosis (MESA) studies [5]. We suggest that differences between the current study and the Beaver Dam study including the participants’ age, angle of fundus photographs, software application for data analyses, and racial composition of the study cohorts contributed to the different findings. Compared to the MESA study, the current study subjects were ethnically homogeneous and the proportion of males was low (37.4% vs 53.6%, respectively). Additionally, our study subjects had lower educational levels, which might also have influenced the results.

A decrease in D(f) was associated with an increase of age in the current study. This finding was also shown in studies using a box counting dimension method to measure D(f) in whites and Malaysians [9], [15]. Another study using Fourier fractal dimension (FFD) techniques to express vascular network complexity also had the same results [36].

### Relationship of retinal vessel parameters with BP

Hypertensive retinopathy is the most common ophthalmologic manifestation of hypertension [37]. Our study results indicated that CRAE was highly correlated with MAP, consistent with numerous studies in different populations including Singaporeans [23], Japanese [38], Caucasians [2], [3], [33], [39], African-Americans [4], and multiethnic populations [5]. MAP showed a stronger association with CRAE than PP, indicating that elevated BP has a stronger impact on central retinal artery stenosis than BP fluctuations. Similar to other studies, this study could not show causality; however, CRAE can predict stroke, mortality of cardiovascular diseases, and coronary heart disease independently of BP levels [39]–[42].

A number of studies [23], [24], [35], [37], [43] have shown that CRVE is not associated with hypertension. The current study also demonstrated no significant association between CRVE and hypertension, but found that a narrow CRVE was not related to an increased PP difference, which is inconsistent with the Rotterdam Study [34]. Increased BP and PP were associated with significant reductions in the retinal arteriolar and venular diameters and the arteriolar-to-venular ratio in the Rotterdam Study. In addition, a study with participants from southwestern Singapore found that retinal arteriolar tortuosity was correlated with elevated BP [44]. PP difference not only reflects a high SBP, but also implies different levels of arterial stiffness. We hypothesize that changes in venules may be due to the same pathological processes resulting in atherosclerosis, i.e., endothelial damage, oxidative stress, and lipid overload.

The current study showed that CRVE was independently associated with uric acid. Uric acid is an important pro-inflammatory factor [45], and some studies have found that uric acid plays a role similar to CRP. Elevated uric acid may suggest occult vasculitis or remodeling of pericapillary spaces [46]. Although the present study could not find the association between CRVE and CRP, other studies have found that increased CRVE was related to specific inflammatory factors. Despite the fact that uric acid is an antioxidant, high uric acid concentrations are associated with microvascular functional reserve or microvascular dysfunction [46]–[48]. This may be related to a series of downstream reactions caused by free radicals generated in the degradation process. Yuan et al. [49] studied a population at high-risk for diabates and found that uric acid concentrations were associated with decreased CRAE and increased CRVE. Our study was a population-based cross-sectional survey and there were methodological differences between the current study and that of Yuan et al.

Studies conducted in whites and Malaysians [9], [15] showed that D(f) was independently associated with SBP, DBP, MABP, and PP. Compared to the association between CRAE and SBP, D(f) showed a stronger association with SBP. The current study found D(f) to be highly correlated with BP classification (prehypertension, hypertension). We speculate that D(f) may have greater sensitivity than CRAE for hypertension morbidity and severity.

### Relationship of retinal vessel parameters and FPG, Hba_1c_, and diabetes

A study in northwest Shanghai reported that participants with narrower retinal arteriolar calibers were diagnosed with metabolic syndrome nearly twice as often as their normal counterparts (odds ratio 1.78) [50]. A relationship of larger CRVE with high HbA_1c_ has been demonstrated [2]–[5], [33]. In addition, Tsai et al. [51] reported that in diabetic patients a wider CRAE was correlated with increasing glucose (*P*<0.001) and HbA_1c_ levels (*P*<0.001). Other studies have found correlations between increasing Hba_1c_ levels, plasma glucose levels, and retinal venular caliber measurements [52]. It is likely that we did not find an association with these measurements due to the differences in our study population compared with previous studies.

To the best of our knowledge, D(f) independently associated with HbA_1c_ has not been reported in other studies: however, this finding might help to explain the association between D(f) and diabetic retinopathy [16], [17], [53]. We suggest that the incidence of low D(f) and diabetic retinopathy is increased in diabetic patients with long-term poor control of blood sugar. Thus, we hypothesize that D(f) might be considered as a predictor of diabetic microangiopathy and target organ damage in diabetic patients.

### Study strengths and limitations

Strengths of the current study are the large number of patients and the fact that it was restricted to the Chinese Han population in coastal regions. Limitations are the study design, which cannot determine causality, selection bias because a relatively large number of subjects were excluded, and patients with ophthalmic diseases such as refractive errors and lens opacity were excluded, which may have influenced D(f). Genetic factors, hormone levels, cell factors, and environmental influences which may have affected retinal vascular parameters were not evaluated. Some bias may be present because retinal vessel diameters are affected by the cardiac cycle.

## Conclusions

In summary, CRAE is predominantly related to gender, smoking, and BP. D(f) was associated with aging, hypertension, and HbA_1c_ level, and D(f) had a stronger association with MAP, SBP, and PP than CRAE.
